# Challenges in school-to-work transition for marginalized groups - the need to strengthen structures for vulnerable youth (post pandemics) Findings from the German project “Co*Gesund”

**DOI:** 10.1093/eurpub/ckac129.453

**Published:** 2022-10-25

**Authors:** M Kuchler, K Heid, A Rademaker, E Quilling

**Affiliations:** 1 Department of Applied Health Sciences, HS Gesundheit Bochum, Bochum University of Applied Sciences, Bochum, Germany; 2 Faculty of Social Sciences, FH Bielefeld, University of Applied Sciences, Bielefeld, Germany

## Abstract

**Objective:**

The transition from school to work is a challenge for young people which is closely correlated with health and well- being. Promoting resilience factors contributes to adolescents’ mental health and social and educational success. The aim of this study is to examine how especially vulnerable youth cope with the transition during the pandemic to identify which structural measures are helpful and which are a hindrance and to develop recommendations for action.

**Methods:**

First, a rapid review on the subject of young people's mental health during the pandemic was conducted. Based on this 30 interviews with professionals from schools and vocational education and two focus groups with young people from participating institutions follow in spring and summer 2022. Finally, recommendations for action will be developed together with both groups.

**Results:**

The results of the literature research illustrate the strong influence of the pandemic on the mental health of adolescents and, in particular, the increase in health inequalities along the social gradient. In relation to school and education, it is clear that young people lacked contact persons during this time. It is expected that the interviews and focus groups give insight, which resources of the young people can be strengthened in a low- threshold way and will provide further concrete indications on what structural development is needed to strengthen the resilience of young people.

**Conclusions:**

For the promotion of mental health it is necessary to rely not only on the resources of young people themselves, but especially on the resources available in their environment. Only in this way transition processes can be managed successfully by the young people and they can be strengthened for the future. To this end, solutions should be developed jointly and support systems should be improved

